# Isolation and Production of Nanocrystalline Cellulose from Conocarpus Fiber

**DOI:** 10.3390/polym13111835

**Published:** 2021-06-01

**Authors:** Anish Khan, Mohammad Jawaid, Lau Kia Kian, Aftab Aslam Parwaz Khan, Abdullah M. Asiri

**Affiliations:** 1 Center of Excellence for Advanced Materials Research, King Abdulaziz University, P.O. Box 80203, Jeddah 21589, Saudi Arabia; draapk@gmail.com (A.A.P.K.); aasiri2@kau.edu.sa (A.M.A.); 2 Chemistry Department, Faculty of Science, King Abdulaziz University, P.O. Box 80203, Jeddah 21589, Saudi Arabia; 3 Laboratory of Biocomposite Technology, Institute of Tropical Forestry and Forest Products (INTROP), Universiti Putra Malaysia, Serdang 43400, Selangor, Malaysia; laukiakian@gmail.com

**Keywords:** nanocrystalline cellulose, conocarpus fibre, morphology, crystallinity, thermal behavior

## Abstract

Conocarpus fiber is a lignocellulosic biomass rich in cellulose potentially used for producing nanocrystalline cellulose (NCC), a biomaterial extensively employed in various application fields. In the present work, different hydrolysis times of 10, 20 and 30 min were applied to chemically pre-treated Conocarpus fiber to produce CPNC1, CPNC2, and CPNC3 particles. With acid hydrolysis treatment, the yield of NCC product was successfully retained at 17–19%. Individual, rod-like shapes of NCC particles could be clearly observed under microscopy examination. From chemical composition analysis, a relatively pure cellulose compartment was produced for all NCC samples with substantial removal of lignin and hemicellulose. The physicochemical analysis proved that each nanoparticle sample possessed strong cellulose crystalline structure. For thermal analyses, the heat resistance of NCCs was gradually enhanced with the increased hydrolysis times. Therefore, the extracted NCC product from Conocarpus fiber could be a green nano-filler for developing nanocomposite material in the future.

## 1. Introduction

Exploitation of new bio-based materials for versatile application fields has received great attention from scientist and researcher to alleviate the climate change through reduced carbon footprint. Biomass wastes are easily available and have become the abundant source for cellulose [1,2,3]. The extensive use of this biomass for manufacturing valuable product, could promote a cleaner and sustainable environment [4,5]. Conocarpus *lancifolius* plant is natively found in Yemen and Somalia countries. Nowadays, it is commonly grown across the North-East Africa, Arabian Peninsula, and also along the coastal lines of tropical regions in North and South America [6,7]. This plant is generally cultivated for ornamental and landscaping purpose in municipal area. However, biomass waste disposal issue may occur from this plant due to improper conduct [8,9]. The Conocarpus trunk part fibre contains great amount lignocellulosic components and its structural built-up is closely similar to the reported study on Ficus *natalensis* plant [10], which serve as major cellulose source. This imparts it with enormous potential for making cellulose derivative product such as microcrystalline cellulose (MCC), cellulose nanowhisker (CNW), cellulose nanocrystal (CNC), and nanocrystalline cellulose (NCC) [11,12,13].

Nanocrystalline cellulose (NCC) is a needle-like shape of cellulose crystallite which produced via inorganic acid hydrolysis. Generally, it has a diameter of 5-30 nm and length of 100-500 nm, with appealing properties like great reinforcing capability, good thermal stability, biodegradable and biocompatible [14,15]. This endows them with wide applicability in papermaking and behaving as nucleating agent in fabrication process. The structural feature, particle size, thermal tolerance, and crystal rigidity of NCC depends primarily on the feedstock source as well as the process conditions including pre-treatment methods, post-treatment reactions, hydrolysis duration, acids type, acids concentration, and temperature [16,17]. NCC is used as excipient, binder, and adsorbent in cosmetic and pharmaceutical industries. Also, in beverage and food industry, they act as additive, emulsifier, stabilizer, thickeners, fat substitute, anti-caking agents, gelling agents, and suspending agents. Additionally, they are broadly employed as reinforcing filler in bio-composite production fields [18].

In previous work, we had comprehensively studied the different cellulosic fibre parts obtained from Conocarpus plant, regarding to their fundamental properties and characteristics [19]. Meanwhile, the results suggested that the trunk fibre part contained the most cellulose component. Also from literature studies, there is no work reported on using Conocarpus fibre as raw material for NCCs isolation. Hence, in present study, the novelty focuses on the utilization of Conocarpus trunk fibre as a new biomass material for producing NCC particles. We further deal with the extraction process of NCCs from raw Conocarpus fibre by employing different acid hydrolysis reaction times after sequential alkali and bleaching chemical treatments. Characterization was carried out for the obtained NCCs to examine their morphology particle size, elemental composition, dispersion behavior and yield of production. Additionally, physico-chemistry, crystallinity, and thermal stability of NCC particles were also evaluated to fully understand their change of properties.

## 2. Materials and Methods

### 2.1. Materials and Chemicals

Conocarpus trunk fibre was obtained from Riyadh, Saudi Arabia. The fibre was grounded to smaller size form of 0.5–1 cm, and then oven-dried for 24 hrs to remove excess moisture content. Chemicals of sodium chlorite (NaClO_2_), acetic acid (C_2_H_4_O_2_), sodium hydroxide (NaOH), and sulfuric acid (H_2_SO_4_) were procured from R&M Sdn. Bhd., Malaysia.

### 2.2. NCC Preparation

Initially, the ground fibre was cooked in 500 ml of 5% NaOH at 80 °C for 5 h in the aim to swell the lignocellulosic fibre and subsequently remove lignin via solubilization. The NaOH-treated fibre residue was then obtained through nylon membrane filtration with distilled water. Afterwards, the fibre was treated in 500 ml of 2% NaClO_2_ (acidified with 5 ml C_2_H_4_O_2_) at 80 °C for 2 h with constant stirring to solubilize hemicellulose. The NaClO_2_-treated fibre residue was collected by filtration and further washed with distilled water until showing white color. After this, acid hydrolysis was carried out for the fibre using 50 wt% H_2_SO_4_ with different reaction times (10, 20, and 30 mins) at 80 °C to produce NCC particles through cellulose depolymerization process. The resulting solution was then added with 10 times volume of cold distilled water to quench its acidic reaction. Hereafter, it was neutralized to pH 3 through several cycles of centrifugation with repeatedly adding distilled water. The resulting white colloidal suspension was left 1 hr for settling down the residual micro-sized cellulose, whereas the supernatant portion containing pure NCCs was collected for dialysis process. Dialysis was then carried out for 7 days in order to remove the excessive ionic elements of natrium (Na^+^), chloride (Cl^-^), chlorite (ClO^2-^), and acetate (CH_3_COO^-^) which generated during chemical treatments. Finally, the suspension was freeze-dried to obtain NCC solid product. The NCC samples isolated with different hydrolysis times of 10, 20, and 30 mins were designated as CPNC1, CPNC2, and CPNC3, respectively.

### 2.3. Characterization

#### 2.3.1. Morphology, Particle Size, Elements, Dispersion Behavior and Yield Analyses

Transmission Electron Microscope (TEM) was applied through FEI Tecnai F20 (Thermo Fisher Scientific Inc., Waltham, MA, USA) to examine the nanostructure of NCC particles. The specimen was prepared by staining the samples with 2% uranyl acetate and deposited on copper grid prior to viewing. Besides this, their surface feature after freeze-drying was examined with Field Emission Scanning Electron Microscope (FESEM) using FEI Nova NanoSEM 450 (Thermo Fisher Scientific Inc., Waltham, MA, USA), which operated under 1–15 kV accelerating voltage. Prior to viewing, samples were coated with platinum metal layer before loading on carbon-taped stub. The size of particle was determined for the nanoparticles via ImageJ software analysis. Elemental composition for each NCC sample was also investigated through Energy Dispersive X-ray (EDX) analysis (Thermo Fisher Scientific Inc., Waltham, MA, USA). Meanwhile, the dispersion behavior was studied with zeta potentials by Dynamic Light Scattering (DLS) using an equipment of Malvern Zeta sizer Nano ZS (Malvern Panalytical B.V., Brighton, UK). Additionally, the chemical composition of each treated fibre sample, including NCCs was analyzed to determine their contents of α-cellulose with TAPPI T203cm-99, holocellulose with TAPPI T249-75, and lignin with TAPPI T222 om-88, whilst the hemicellulose was determined by deducting with α-cellulose from holocellulose. The yield (%) of each fibre sample production was also calculated with the Equation (1) provided as below:

(1) $$Yield\;\left(\%\right)=\frac{\left(M_{2}-M_{3}\right)}{M_{1}}\times 100\%$$

in which *M_1_* represents mass of raw Conocarpus fibres; *M_2_* represents total mass of treated-fibres within weighing container; *M_3_* represents mass of weighing container. Triplicate works were also conducted for the chemical composition and yield production to obtain reproducibility proof.

#### 2.3.2. Chemical Functionality Analysis

The functional groups of NCCs were investigated using Thermo Nicolet Nexus 670 Fourier Transform Infrared Ray (FTIR) analyzer (Thermo Fisher Scientific Inc., Waltham, MA, USA) with wavenumbers ranging 4000–400 cm^−1^ maintained at 4 cm^−1^ resolution. Before analysis, the samples were grinded with potassium bromide (KBr) and then underwent pelletization.

#### 2.3.3. Crystalline Analysis

A PANalytical Empyrean X-ray diffractometer (XRD) (Malvern Panalytical B.V., Brighton, UK) was used to study the crystallinity of NCCs. The X-ray generator operated at 45 kV and 40 mA with Cu Kα radiation in 2°/min scan rate, while the powder samples were placed on nickel coated steel holder.

#### 2.3.4. Thermal Stability Analysis

Thermal behavior of NCCs was evaluated with TA-SDT Q600 thermo gravimetric analyzer (Mettler-Toledo International Inc., Columbus, OH, USA). Both analyses of Thermogravimetry (TGA) and Derivative Thermogram (DTG) were conducted in 30–900 °C temperature range at 10 °C/min heating rate under nitrogen purge atmosphere. Meanwhile, Differential Scanning Calorimetry (DSC) (Mettler-Toledo International Inc., Columbus, OH, USA) was run in 30–600 °C temperature range with 10 °C/min heating rate. 

## 3. Results and Discussion

### 3.1. Morphology, Particle Size, Chemical Composition and Yield

**Figure 1:**
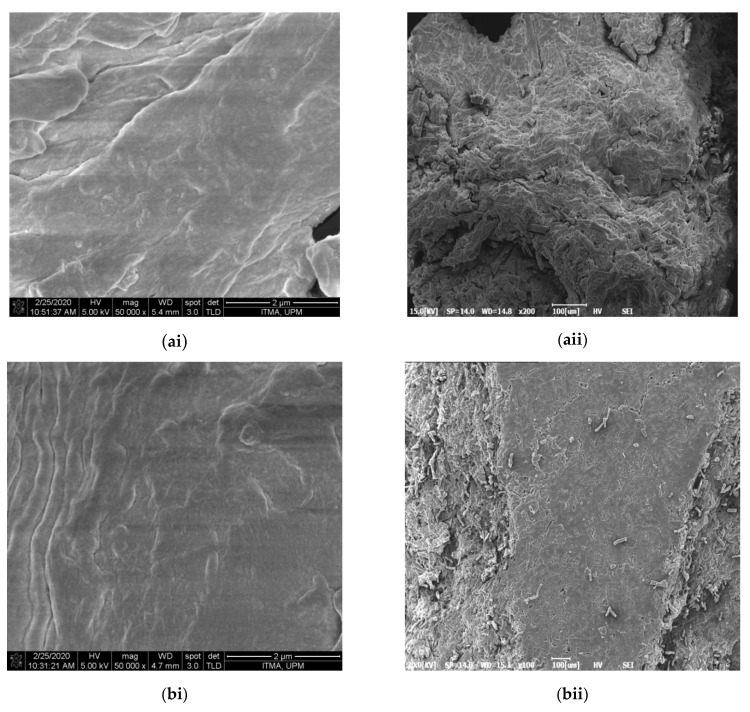

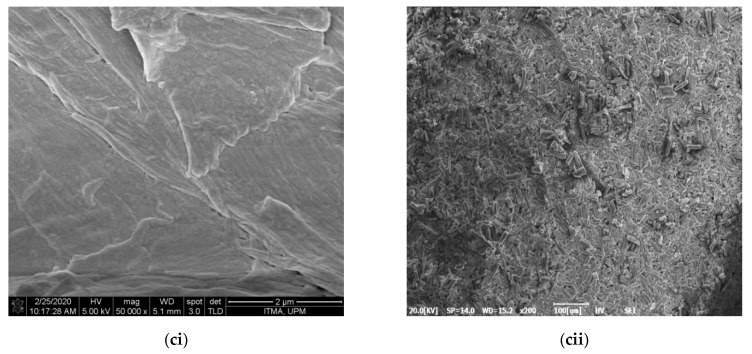
FESEM images of (**a**) CPNC1, (**b**) CPNC2, and (**c**) CPNC3 under ×1000 (**i**) and ×50,000 (**ii**) viewing.

Figure 1 displays the morphology of NCC samples under FESEM examination. With surface viewing (Figure 1), CPNC1 showed bundle-like features with rough surface due to the undisintegrated fibril structure. For CPNC2 and CPNC3 samples, they exhibited relatively smooth surfaces, indicating the nanoparticles were well separated from the bulky fiber. These small size particles were able to arrange symmetrically with each other and formed flat surface [20]. Under magnified viewing (Figure 1), a large bundle feature was revealed by CPNC1, indicating the fibrous structure still remained intact in this short period of reaction time. With increased hydrolysis time, short nanocrystallite particles prevailed in the CPNC2 samples, showing the fiber disintegration process began at this stage for isolating NCC particles [21]. Moreover, the CPNC3 sample presented more individually segregated nanoparticles, showcasing the hydrolysis reaction was optimized in this circumstance [22].

**Figure 2:**
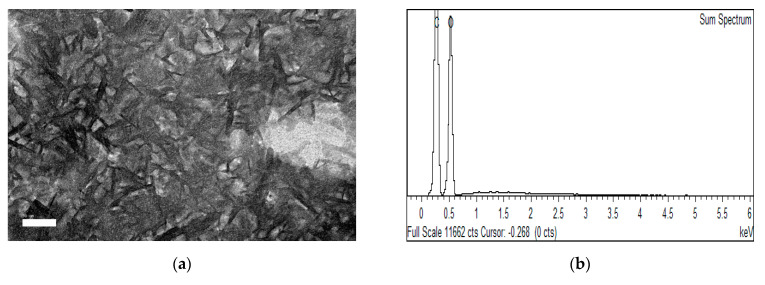

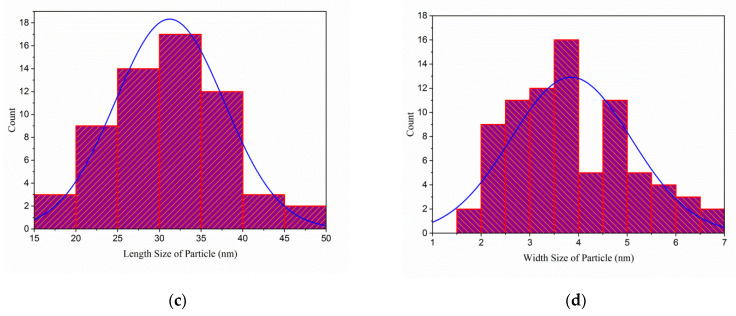
Images of (**a**) TEM, (**b**) EDX, (**c**) width size distribution, and (**d**) length size distribution for CPNC3 sample.

From TEM examination, significant amounts of rod-like shapes of nanocrystals were observed for CPNC3 (Figure 2a). Meanwhile, the particle size analysis formed normal distribution curves, as shown in Figure 2c,d, respectively, proving the isolated CPNC3 particles had considerably uniform sizes. The estimated average size for CPNC3 sample was 3.85 nm in width and 31.23 nm in length. As well with its high aspect ratio of about 8.1, it could be favorable for high surface reactivity when processing material fabrication [11,23]. From EDX analysis (Figure 2b), CPNC3 revealed carbon and oxygen as the major elements in the composition, implying the typical characteristic of NCC structure. Additionally, none of the other impurities were traced in the analysis, and this evidenced the produced NCC particle was pure for the CPNC3 sample [24,25]. This was in line with the improvement of α-cellulose content (to about 80–85%) for NCCs sample treated with increased hydrolysis times, as shown in Table 1. Substantial amounts of residual hemicellulose and lignin components were removed at about 45–60% for both CP-NaOH and CP-NaClO_2_ samples due to chemical solubilization. Further, some of the remained residues were eliminated via acidic degradation during hydrolysis for NCCs isolation [26]. Nevertheless, the yield of NCCs produced in this work was considerably high at between 17.6% and 18.9%, suggesting the Conocarpus fiber had great economic feasibility.

### 3.2. FTIR

**Figure 3:**
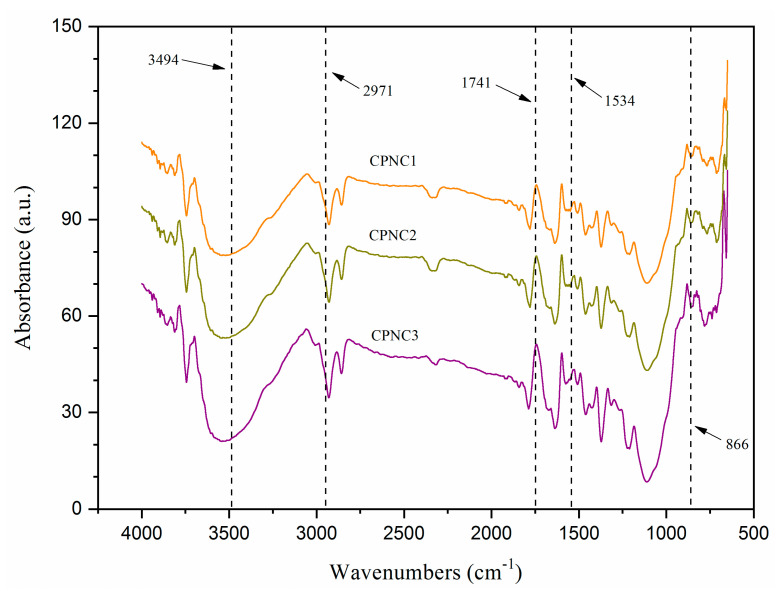
FTIR spectra of CPNC1, CPNC2 and CPNC3.

Figure 3 displays the FTIR spectra of each NCC were similar, showing the chemical groups were not modified with various hydrolysis times [22]. A broad absorption peak at about 3494 cm^−1^, representing the -OH vibration groups of cellulose, was observed in all samples. This peak was more intense from CPNC1 to CPNC3, indicating the cellulose order changed via the different hydrogen bonding interaction [24]. Meanwhile, another peak at around 2971 cm^−1^ was assigned to the C-H bending. It became sharper from CPNC1 to CPNC3, revealing the improved exposure of cellulose component [26]. Besides this, there was a notable 1636 cm^−1^ peak with its shoulder band at about 1692 cm^−1^, proving those samples possessed hydrophilic property for interacting water molecules [23]. The residual components such as hemicellulose and lignin were also substantially removed from all samples with the absence of peaks at 1741 cm^−1^ (ester group stretching) and 1534 cm^−1^ (C=C aromatic vibration), respectively [27]. Furthermore, the “crystallinity peaks’” at 1425 cm^−1^ (symmetric CH_2_ stretching) and 1464 cm^−1^ (asymmetric CH_2_ stretching) increased in intensities for CPNC3, implying the enhanced crystallites structure within cellulose [20,28]. Additionally, the anhydroglucose-related peaks at 1373 cm^−1^ (C–H asymmetric stretching), 1092 cm^−1^ (C–O–C pyranose ring), and 865 cm^−1^ (β-1,4-glycosidic linkage) were observed for each sample, signaling the typical functional chemistry of NCC product [29].

### 3.3. XRD

**Figure 4:**
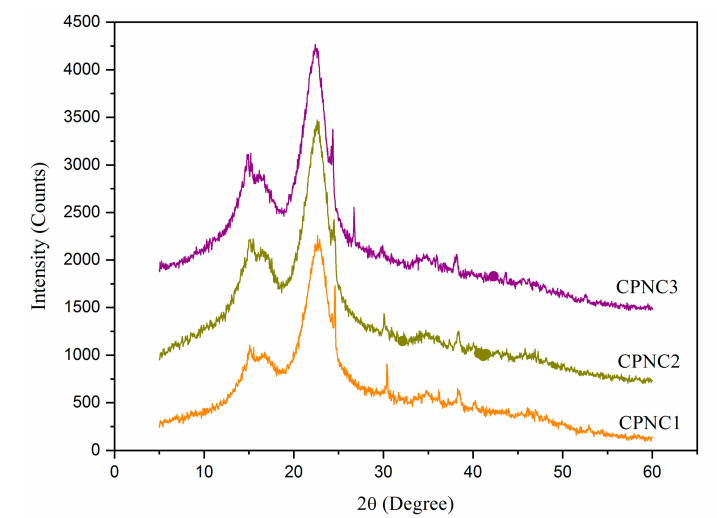
XRD spectra of CPNC1, CPNC2, and CPNC3 samples.

XRD diffractogram of NCCs is shown in Figure 4. All samples showed pronounced peaks at 15.2°, 16.7°, 22.6°, and 34.5°, which correlated, respectively, to (1–10), (110), (200), and (004) crystallography planes of Iβ cellulose polymorph structure [28,29]. The peak intensity at 22.6° was greatly enhanced for both CPNC2 and CPNC3 samples, indicating the cellulose crystalline region was predominant in the sample [20,27]. Meanwhile, the peaks at 15.2° and 16.7° were noticed to be changing in intensities for each sample. It is possible the prolonged hydrolysis times promoted the acidic penetration that somehow affected the internal cellulose organization [25]. In addition, another crystalline peak at about 34.5° showed slightly increased sharpness from CPNC1 to CPNC3, which was likely contributed by the enhanced cellulose crystal order [3,22]. Moreover, the determined crystallinity degrees were the highest with 79.3% for CPNC3, followed by 78.2% for CPNC2 and 75.3% for CPNC1.

### 3.4. Thermal Analysis

**Figure 5:**
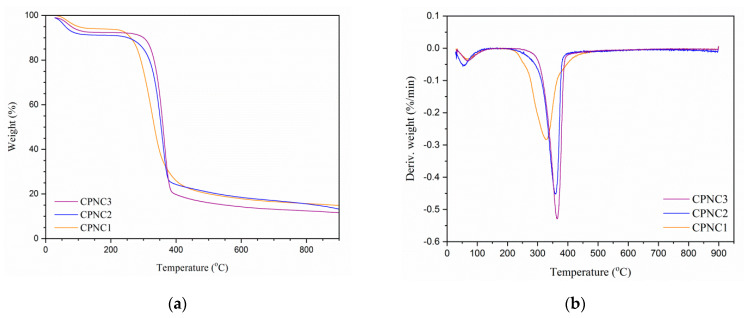
(**a**) TGA and (**b**) DTG spectra of CPNC1, CPNC2, and CPNC3 samples.

Thermogravimetric analysis is illustrated in Figure 5, and its data are summarized in Table 2. At beginning, each sample showed weight loss in the temperature profile of 80–130 °C as a result of the volatized water content [20]. Beyond 200 °C, the onset decomposition temperature was tremendously increased from CPNC1 (at 266.3 °C) to CPNC3 (at 324.7 °C), implying the thermal resistance of nanoparticles was improved with longer hydrolysis times [24]. Between 300–400 °C, the maximum weight loss increased for the CPNC3 sample with 76.7%, showcasing the uniform decomposition process of cellulose. At the meantime, its reduced residual formation also indicated the isolated nanoparticles were pure [28]. Furthermore, CPNC2 and CPNC3 presented similar patterns of TGA spectra, indicating they had the same thermal degradation behavior. It was supported by the DTG spectra where both samples had nearly the same peak decomposition temperatures at around 362.0 °C [25]. In addition, CPNC3 also revealed the highest peak decomposition temperature at 364.5 °C, probably due to its compact cellulose crystals arrangement compared to other samples [26].

**Figure 6:**
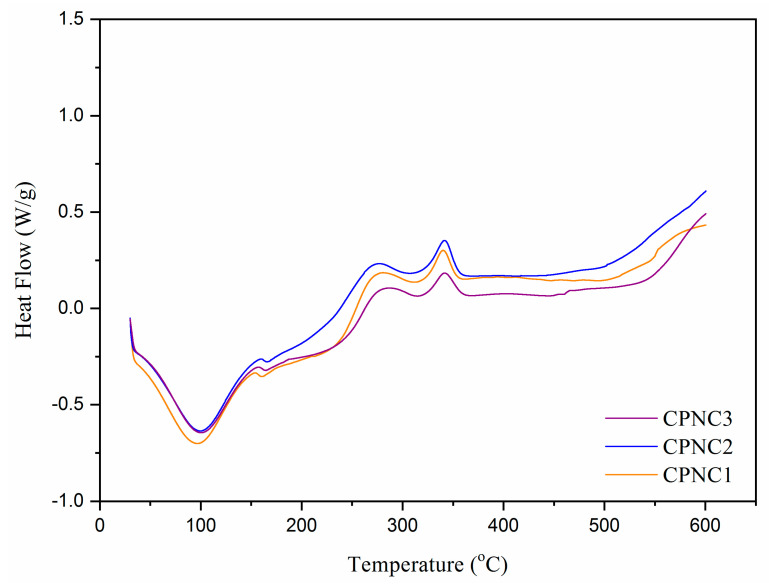
DSC spectra of CPNC1, CPNC2 and CPNC3 samples.

The thermo-molecular behavior within NCC samples was analyzed with DSC spectra, as shown in Figure 6. Heat energy was absorbed initially by nanoparticles to evaporate the remaining water content, where the large endothermic bands were shown in the region of 50–130 °C [16]. In between 150–200 °C, a small endotherm was noticed for each NCC sample, which related to the absorption of heat for cellulose decarboxylation. When compared to CPNC1 (at 160.3 °C), this endothermic peak shifted to the right for both CPNC2 (at 165.5 °C) and CPNC3 (at 164.2 °C), probably due to the predominance of cellulose crystallite feature [29]. From 330 °C to 370 °C, an exothermic peak associated with the cellulose decomposition was noticed for those samples. This peak shifted right for CPNC3 (at 342.6 °C), proving the thermal decomposition of cellulose was more stable for the sample as compared to others [30]. Beyond 400 °C, all samples ultimately were subjected to liquefaction and gasification processes [31,32].

## 4. Conclusions

The present study revealed the findings of utilizing different hydrolysis times for isolating NCC products from Conocarpus fiber. From morphology examination, individually well dispersed NCC particles with rod-like shapes were successfully obtained throughout the chemical treatments. The isolated NCC particles contained pure cellulose elements composition and less impurity, giving them the potential to be employed in food, pharmaceutical, and biomedical applications. Meanwhile, the crystalline structure of NCC particles was insignificantly affected by the increased hydrolysis times. In addition, high thermal resistance property was also possessed by those NCC particles, endowing them with suitability to be applied in extreme temperature processing conditions. Hence, the Conocarpus fiber could be a promising alternative biomass for manufacturing NCC products in the future.

## Figures and Tables

**Table 1:** Chemical composition and yield of different fibres.

Samples	α-Cellulose (%)	Hemicellulose (%)	Lignin (%)	Yield (%)
CP-raw	41.7 ± 0.32	28.5 ± 0.08	15.6 ± 0.02	-
CP-NaOH	69.3 ± 0.51	15.5 ± 0.19	9.3 ± 0.05	56.6 ± 0.37
CP-NaClO_2_	70.2 ± 0.54	14.1 ± 0.17	7.5 ± 0.04	51.9 ± 0.41
CPNC1	81.6 ± 0.67	6.1 ± 0.22	2.9 ± 0.11	17.6 ± 0.27
CPNC2	82.3 ± 0.66	5.8 ± 0.24	2.6 ± 0.13	18.3 ± 0.29
CPNC3	84.9 ± 0.64	5.3 ± 0.18	2.2 ± 0.09	18.9 ± 0.34

**Table 2:** Thermal data of CPNC1, CPNC2, and CPNC3 samples.

Samples	O_dT_ (°C) ^a^	P_dT_ (°C) ^b^	M_WL_ (%) ^c^	R_W_ (%) ^d^
CPNC1	266.3	327.7	74.2	15.1
CPNC2	304.2	359.0	73.8	14.3
CPNC3	324.7	364.5	76.7	12.8

^a^ onset decomposition temperature; ^b^ peak decomposition temperature; ^c^ maximum weight loss; ^d^ residual weight formation.
